# Challenges and opportunities in obesity: the role of adipocytes during tissue fibrosis

**DOI:** 10.3389/fendo.2024.1365156

**Published:** 2024-04-15

**Authors:** Qian Zhang, Chongxuan Lu, Feng Lu, Yunjun Liao, Junrong Cai, Jianhua Gao

**Affiliations:** ^1^Department of Plastic and Cosmetic Surgery, Nanfang Hospital, Southern Medical University, Guangzhou, Guangdong, China; ^2^The Second School of Clinical Medicine, Southern Medical University, Guangzhou, Guangdong, China

**Keywords:** obesity, fibrosis, adipocytes, fibroblasts, AMT

## Abstract

Obesity is a chronic disease that affects the energy balance of the whole body. In addition to increasing fat mass, tissue fibrosis occurred in white adipose tissue in obese condition. Fibrosis is the over-activation of fibroblasts leading to excessive accumulation of extracellular matrix, which could be caused by various factors, including the status of adipocytes. The morphology of adipocytes responds rapidly and dynamically to nutrient fluctuations. Adaptive hypertrophy of normal adipocytes protects peripheral organs from damage from lipotoxicity. However, the biological behavior of hypertrophic adipocytes in chronic obesity is abnormally altered. Adipocytes lead to fibrotic remodeling of the extracellular matrix by inducing unresolved chronic inflammation, persistent hypoxia, and increasing myofibroblast numbers. Moreover, adipocyte-induced fibrosis not only restricts the flexible expansion and contraction of adipose tissue but also initiates the development of various diseases through cellular autonomic and paracrine effects. Regarding anti-fibrotic therapy, dysregulated intracellular signaling and epigenetic changes represent potential candidate targets. Thus, modulation of adipocytes may provide potential therapeutic avenues for reversing pathological fibrosis in adipose tissue and achieving the anti-obesity purpose.

## Graphical Abstract

Graphical abstract: obesity-induced adipose tissue fibrosis. In chronic obesity, the adipocytes that have undergone excessive expansion demonstrate heightened inflammatory activity, impaired secretion function, and inadequate vascularization, ultimately creating a microenvironment conducive to fibrosis development and leading to the formation of adipose tissue fibrosis.
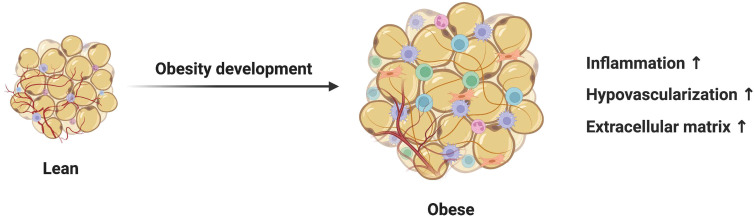

## 1 Introduction

In recent years, the growing prevalence of obesity has become a major public health problem worldwide. As the body’s largest energy store, adipose tissue plays an important role in controlling energy balance throughout the body. Benefiting from the characteristic loose ECM structure, mature adipocytes can support fatty acid release or storage by resizing from small cells with a diameter of 20–70 μm to large cells with a diameter of 300 μm (1). At the same time, adipocytes can secrete many lipid and protein factors through endocrine action, which have a profound impact on the metabolism of other tissues. Recent studies have shown that adipocytes exhibit remarkable plasticity during periods of caloric excess, driving the development of extracellular matrix remodeling. In the early stages of obesity, adipocytes in subcutaneous adipose tissue adapt to increased energy supply by increasing intracellular lipid accumulation and hypertrophy. However, the over-expanded adipocytes in chronic obesity are characterized by up-regulation of inflammatory activity, secretion dysfunction, and abnormal differentiation, inducing a microenvironment conducive to fibrosis (2). Fibrosis is a chronic process of ECM excessive accumulation characterized by hyperactivation of myofibroblasts. The highly rigid ECM in fibrotic adipose tissue induces apoptosis and lipid leakage in normal adipocytes through shear stress, thereby triggering common metabolic syndromes such as dyslipidemia and insulin resistance (3). In addition, elevated concentrations of pro-fibrotic adipokines in circulation have been shown to exert a negative influence on other tissues and organs. The persistent fibrotic response detected in chronic diseases such as cirrhosis, systemic scleroderma, and heart failure is closely related to uncontrolled adipocytes. The medical burden caused by fibrotic diseases is enormous. It is estimated that up to 45% of deaths in developed countries can be attributed to organ failure due to fibrosis-related diseases (4, 5).

The synergistic involvement of myofibroblasts in tissue fibrosis with different types of cells, such as inflammatory immune cells, has been extensively explored. However, little attention has been paid to the contribution of adipocytes in extracellular matrix remodeling. Here, we update our current understanding of adipose tissue fibrotic remodeling in the obese state, focusing on the role of adipocytes. A better understanding of the autocrine, paracrine and endocrine communication mechanisms of adipocytes may provide further insights into the pathobiology of obesity-related fibrotic diseases. Finally, we delve into the challenges and prospects encountered in this field, aiming to stimulate significant research in related domains and provide novel insights for the prevention and treatment strategies of fibrosis-related diseases.

## 2 Adipocytes regulate adipose tissue fibrosis

### 2.1 Adipocytes promote fibrosis by releasing free fatty acids

**Figure 1:**
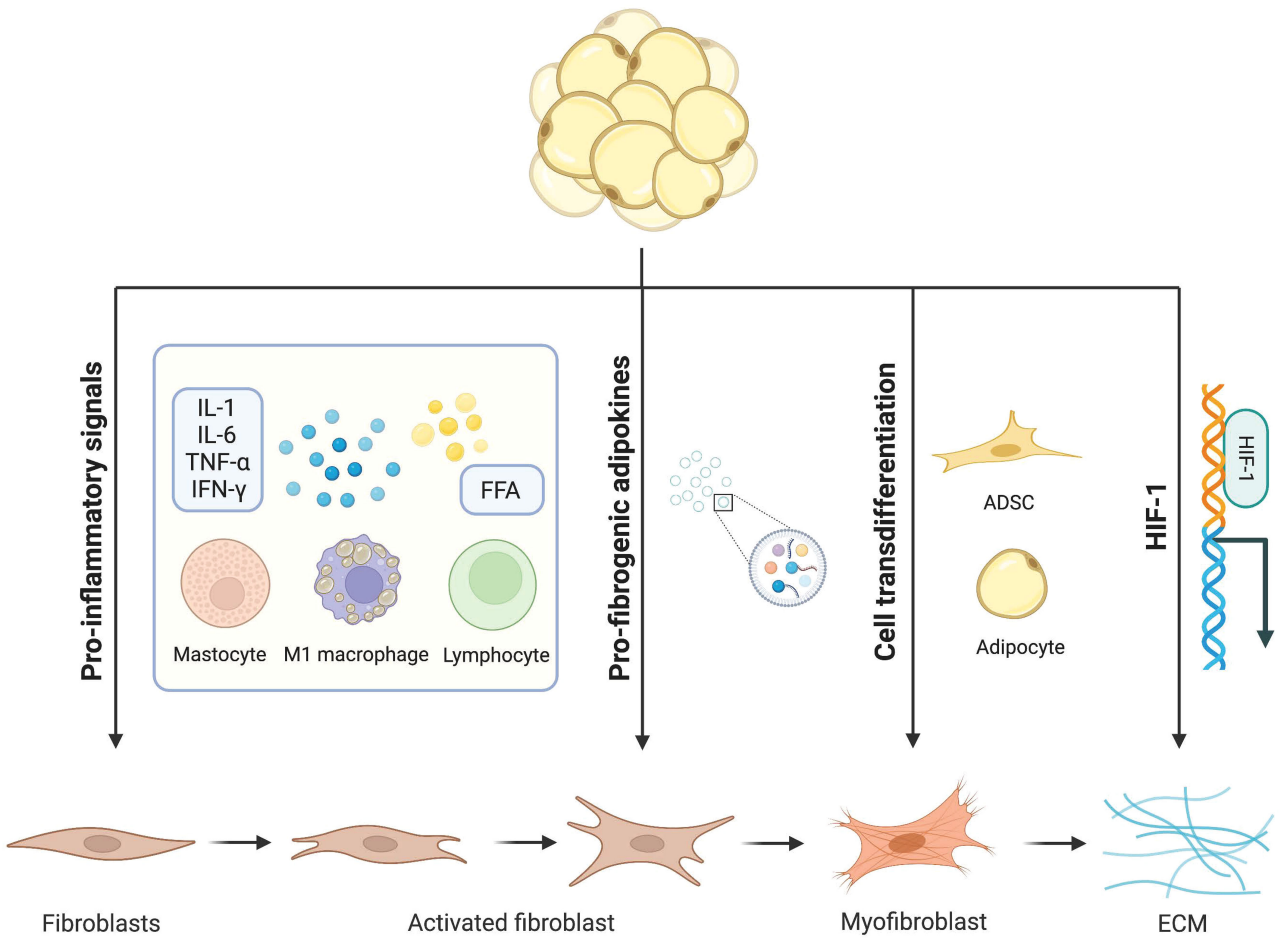
Mechanisms of adipocytes in adipose tissue fibrosis. Dysfunctional adipocytes secrete proinflammatory cytokines and free fatty acids, which activate immune inflammatory cells and amplify the inflammatory cascade. In addition, imbalanced adipokines and hypoxia-inducible factors further promote the development of adipose tissue fibrosis. In this condition, mature adipocytes and adipose stem cells transdifferentiate into myofibroblasts. Activated myofibroblasts aggregate and a large amount of extracellular matrix is deposited in adipose tissue.

In obesity, the inherent ability of adipose tissue to store and sense nutrients is compromised, resulting in free fatty acids (FFA) spilling into the periphery and circulation (**Figure 1**). Miller et al. explored the effect of free fatty acids on adipose tissue function by developing a unique dietary regimen and found out that the peroxidized n-3-enriched diet led to lipotoxicity of white adipose tissue, as evidenced by increased fibrosis, lipofuscin, and reduced anti-inflammatory markers (6). Mechanisms of lipotoxicity involve various cellular processes, including mitochondrial damage, and activation of intracellular inflammation-related signaling pathways (7). Furthermore, signaling pathways related to fatty acid-mediated adipose tissue fibrosis are also particularly enriched. Targeted stimulation of ERK signaling in hypertrophic adipocytes increases free fatty acid production and release from lipolysis, which further upregulates inflammatory pathways in adipocytes (8). Obese adults with low fatty acid prevalence have less adipose tissue fibrosis compared with high fatty acid prevalence, which is associated with lower activation of the SAPK/JNK pathway (9). In addition, the bile acid-activated nuclear receptor, FXR has been implicated in the control of and may be a key determinant of adipocyte size and adipose tissue function under metabolic stress. Sustained FXR expression in adipose tissue limits its storage capacity, leading to elevated plasma-free fatty acids, ultimately promoting adipose tissue fibrosis (10). Besides, lipotoxic cell damage also mediates fibrotic responses in various organs such as the heart and kidneys (11, 12).

### 2.2 Adipocytes contribute to fibrosis via dysregulated secretion of adipokines

Apart from its crucial involvement in lipid metabolism, adipose tissue serves as an essential endocrine organ by releasing a range of specific cytokines and hormones referred to as adiponectins (13). Adipokines interact with receptors on target cells, triggering intracellular signaling pathways and inducing various effects. In cases of obesity-induced disrupted fibrotic adipose tissue, there is an overproduction of dysregulated adipokines that often display proinflammatory and profibrotic characteristics.

#### 2.2.1 Adiponectin

Adiponectin, one of the most abundant circulating adipokines, is a protective protein highly expressed in adipocytes (14, 15). Activation of adiponectin receptors has been shown to exert potent anti-inflammatory and anti-fibrotic effects in nonalcoholic steatohepatitis (NASH) models (16). *In vitro* studies have demonstrated that adiponectin can inhibit fibroblast activation induced by TGF-β, LPS, and Wnt signaling pathways, as well as downregulate collagen and α-SMA gene expression (17, 18). Therefore, adiponectin can be identified as a negative regulator of tissue fibrosis. Unfortunately, obesity significantly reduces the secretion of adiponectin (19). In humans, plasma concentration of adiponectin is negatively correlated with body weight and BMI (20). Furthermore, exercise-induced mitigation of high-fat diet-induced hypertrophy in adipocytes and collagen deposition leads to a significant increase in adiponectin levels within the adipose tissue (21).

#### 2.2.2 Proinflammatory cytokines

Low-grade chronic inflammation caused by adipocytes in obesity is thought to be a key factor in adipose tissue fibrosis. Although increased numbers of adipocytes are well tolerated for obesity, hypertrophy of their size is considered a deleterious process (22). When a certain threshold is reached, anabolic stress causes widespread molecular changes in fat cells. Dysfunctional adipocytes release more pro-inflammatory factors such as tumor necrosis factor-α (TNF-α), interleukin 6 (IL-6) and interleukin 1 (IL-1) (23). Among them, TNF-α and IL-6 are potent agonists of collagen synthesis. Overexpression of TNF-α induced macrophage infiltration and subsequent fibrosis in adipose tissues under the HFD regimen (24). Furthermore, TNF-α promoted the differentiation of mesenchymal stem cells into fibroblasts by activating NF-κB signaling (25). In a model of renal fibrosis, specific blockade of IL-6 signaling reduced the number of phosphorylated signal transducers and activators of transcription (p-STAT3)-activated fibroblasts and the deposition of extracellular matrix proteins (26). As for changes in inflammatory fatty acid metabolism, they indirectly modulate fibrosis by increasing lipotoxicity or altering cell fate (27).

#### 2.2.3 Hypoxia-inducible factor

WAT is one of the most vascularized tissues in the body. Angiogenesis is the physiological process of forming new blood vessels based on existing blood vessels, which is essential for the maintenance of normal tissue physiological function and tissue remodeling. With further lipid accumulation, the density and function of intrinsic capillaries cannot support the massive expansion of adipose tissue and hypoxia ensues (28). On the one hand, hypoxia indirectly promotes fibrosis by activating the inflammatory response. The necrosis of fat cells caused by hypoxia is conducive to the infiltration of pro-inflammatory leukocytes. Studies have also shown that hypoxia can alter the adipose-derived mesenchymal stem cells (ADSCs) secretion profile of (METS) patients to release more pro-inflammatory factors and trigger a specific inflammatory state (29). On the other hand, both the stability and transcriptional activity of the hypoxia-inducible factor(HIF-1) were increased in adipocytes and further activated the pro-fibrotic transcriptional program under hypoxia (30). AT of transgenic ob/ob mice expressing HIF-1 exhibit increased fibrosis and upregulation of several ECM genes (31). HIF-1 promoted the accumulation of type I collagen in response to transforming growth factor-β (TGF-β) signaling by forming a transcriptional complex with small mothers against decapentaplegic 3 (Smad3) (32). At the same time, studies have also shown that activation of HIF-1 signaling can promote epithelial-mesenchymal transition (EMT), thereby exacerbating fibrotic damage (33). Moreover, selective inhibition of HIF-1 reduces adipose tissue inflammatory infiltration and tissue fibrosis in high-fat diet mice (34).

#### 2.2.4 Visfatin

Visfatin is highly enriched in visceral adipose tissue, and its expression level in plasma increases with the development of obesity (35). Treatment of 3T3-L1 preadipocytes with visfatin resulted in up-regulation of adipose tissue fibrosis markers such as collagen type VI (Col6) (36). In addition, visfatin accelerated the development of liver fibrosis by increasing the expression of α-SMA, fibronectin, vimentin, and CTGF as well as inflammatory chemokines (37).

#### 2.2.5 HMGB1

The novel adipokine high mobility group box 1 (HMGB1) is a 30 kDa DNA-binding protein, which is an important regulator of extracellular matrix remodeling. HMGB1 was present in a variety of cells but mainly secreted by adipocytes in visceral adipose tissue in obese patients (38). As an autocrine medium, HMGB1 in the extracellular environment has a certain pro-inflammatory effect on human adipocytes, which increased the expression of toll-like receptor 4 (TLR4) and toll-like receptor 2 (TLR2) as well as promoted the infiltration of M1-type macrophages in adipose tissue through the NF-κB signaling pathway (39). In addition, HMGB1 increased the release of active TGF-β1 in macrophages and fibroblasts to accelerate the transformation of fibroblasts into myofibroblasts (40). However, no direct link between HMGB1 and adipose tissue fibrosis has been reported, and further research is needed in the future.

#### 2.2.6 Leptin

Leptin is a small peptide derived from adipose tissue. During normal physiology, leptin crossed the blood-brain barrier and inhibited the secretion of neuropeptide Y and agoutin to reduce hunger and increase energy expenditure. Leptin also bonds directly to leptin receptors and inhibited lipid synthesis in adipocytes (41). Obesity-related hyperleptinemia may be another cause of fibrosis. Leptin has been shown to induce proadipogenic and proinflammatory Signaling in adipocytes and ASCs by activating the mTOR pathway (42). The propelling role of leptin in adipose tissue fibrosis has been further confirmed. Leptin activated TGF-β and CTGF through the phosphatidylinositol 3 kinase (PI3K)- protein kinase B (AKT) signaling pathway to induce collagen deposition and promote fibrosis (43, 44). In addition, leptin-mediated aldosterone production may be a new mechanism for obesity-related fibrosis (45).

#### 2.2.7 DPT

Dermatopontin (DPT), also known as tyrosine-rich acidic mechanism protein (TRAMP), can promote collagen accumulation to some extent. However, the secretion of DPT from visceral adipose tissue was greatly increased in the obese state, which exacerbated the remodeling of the extracellular matrix and the occurrence of chronic inflammation in adipose tissue (46).

#### 2.2.8 Endotrophin

A novel adipocyte-derived factor endotrophic is the C-terminal cleavage product involving this C5 domain of recombinant collagen type-VIα3 (COL6α3). The expression of endotrophic is mainly derived from fully differentiated adipocytes and is hardly in ADSCs. Its levels are upregulated in obese individuals (47). Endorphins can act as powerful co-stimulators of existing pathological processes in “unhealthy” adipose tissue, triggering further enhancement of fibrosis and inflammation when challenged with a high-fat diet (HFD). Profibrotic and proinflammatory genes were significantly upregulated in the fat pad of Endotrophin-overexpressing transgenic mice. Furthermore, in a diet-induced obesity (DIO) model, transgenic mice efficiently induced a fibrotic microenvironment in adipose tissue by upregulating collagens, collagen cross-linking enzymes lipoxygenase (LOX), and additional ECM constituents. Besides, some of these fibrotic actions may be exerted through upregulation of the TGF-β pathway (48).

### 2.3 Adipocytes promote fibrosis by extracellular vesicle delivery

Extracellular vesicles (EVs) have been identified as a novel mode of communication between different cells and tissues. Intracellular signaling depends on the functional molecular composition of EVs, reflecting the physiological state of producing cells and tissues (49). Mature adipocytes secrete more EVs under the challenge of metabolic stress, elevating the level of circulating EVs (50, 51). Recently, an emerging role of adipocyte-derived EVs in obesity-related fibrotic comorbidities has been recognized. Obesity altered the secretion profile of functional miRNAs in adipocyte-derived EVs. MiRNAs involved in pro-inflammatory signaling and programmed cell death were up-regulated, and those involved in anti-fibrotic and angiogenic pathways were excluded (52, 53). Not only that, EVs released from adipocytes derived from obese patients are enriched in different proteins involved in ECM remodeling to promote adipose tissue fibrosis during obesity, including collagens, metalloproteinases, and ECM receptors (54).

### 2.4 Adipose cells promote fibrosis by activating immune cells

Adipose tissue is recognized as a natural reservoir of inflammatory cells, including macrophages, natural killer cells, mast cells, eosinophils, and lymphocytes (55). Inflammatory cells are mainly responsible for maintaining immune homeostasis under physiological conditions. However, in the obese adipose tissue microenvironment, adipocytes secrete inflammatory factors to recruit and activate surrounding inflammatory cells. Crosstalk between adipocytes and inflammatory cells further promotes the process of fibrosis. For example, the levels of Adipocyte-secreted exosomal microRNA-34a (miR-34a) were elevated in obesity compared to normal, which favored the infiltration of M1 macrophages (56). A unique structure called a crown-like structure (CLS) is formed by macrophages to clear necrotic fat cells. Macrophage-inducible C-type lectin (Mincle) is localized in CLS and promotes the expression of fibrosis-related genes, thereby leading to myofibroblast formation possibly through intercellular communication between macrophages and fibroblasts (57). Furthermore, Adipocytes induced with macrophage-derived medium from obese adipose tissue significantly reduced the expression of adipogenic genes such as peroxisome proliferator-activated receptor-γ (PPAR-γ), while overexpression of inflammatory and extracellular matrix synthesis genes (58). Similarly, white adipose tissue (WAT) from obese humans and mice contains more mast cells than from lean individuals (59). Mast cells adhere and activate fibroblasts in a PAI1-dependent manner, culminating in a cascade of events leading to fibrogenesis (60). At the same time, it has also been shown that the amount of Group 1 innate lymphoid cells (ILC1s) is increased in obese T2D patients and induces adipose fibrosis by releasing IFN-γ (61).

### 2.5 Adipocytes promote fibrosis by downregulating adipogenic differentiation of adipose precursor cells

In addition to differentiated mature adipocytes, adipose tissue also contains various other cell types. Among them, the most extensively discussed are the adipose precursor cells located in the stromal vascular fractions (SVFs) of adipose tissue. Adipose precursor cells from different reservoirs exhibit significant phylogenetic differences and heterogeneity, as well as varying differentiation potential (62). Under normal physiological conditions, these adipose precursor cells maintain a high degree of self-renewal and proliferation to support the homeostasis and expansion of adipose tissue (63). However, obesity impairs the immunoregulation and survival efficacy of ADSCs. ADSCs derived from obese individuals demonstrate impaired anti-inflammatory phenotypes (64). When exposed to an excessive accumulation of inflammatory cytokines in their microenvironment, stem cell transformation and proliferation abilities become compromised, leading to premature cellular decline (65). Additionally, aging ADSCs not only exhibit down-regulated ability for adipogenic differentiation but also show significant enrichment in genes related to collagen production and inflammatory function (66). Studies have further revealed that with increasing BMI levels, there is upregulation of IL-1R-like 1 expression in mature adipocytes which can inhibit the differentiation of adipose precursor cells thereby reducing adaptive expansion capacity within the adipose tissue (67).

### 2.6 Adipocytes promote fibrosis by transdifferentiation into myofibroblasts

**Figure 2:**
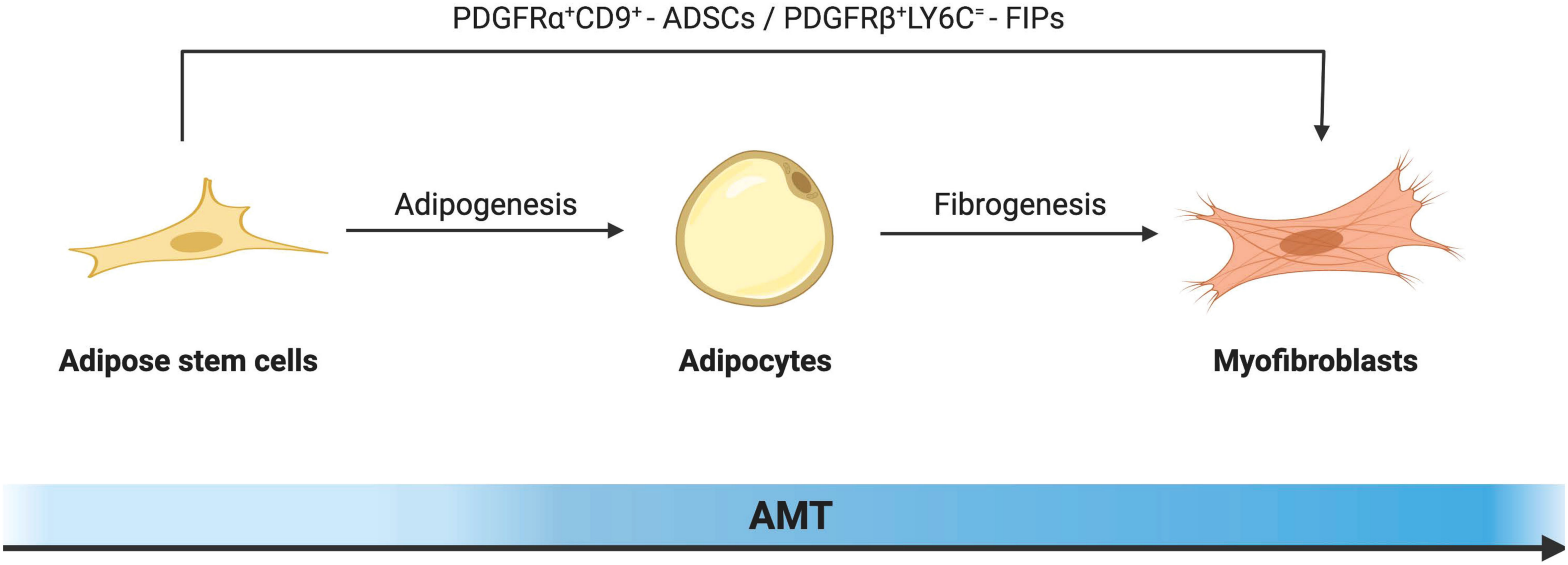
Schematic diagram illustrating the cellular mechanism of adipocyte-mesenchymal transition (AMT) in adipose tissue remodeling under the challenge of obesity. In response to a high-fat diet, alongside the classical fibroblast activation pathway, highly plastic adipocytes can also undergo transdifferentiation into myofibroblasts. By downregulating key adipogenic gene markers including PPAR-γ, both mature adipocytes and adipose stem cells initiate the fibrosis pathway as the predominant mechanism, leading to morphological alterations and gene reprogramming that drive their differentiation towards a myofibroblast phenotype.

Fibroblasts and myofibroblasts are the major producers of adipose tissue fibrosis (**Figure 2**). Myofibroblasts possess both the ECM synthesis capacity of fibroblasts and the cytoskeletal contractility of smooth muscle cells. This enables myofibroblasts to exert pressure on the ECM, thereby activating and releasing latent TGF-β within it, exacerbating the tissue fibrosis process (68). The traditional view of cell differentiation holds that cells follow a defined differentiation trajectory during development, starting with stem cells and ending in a terminally differentiated state. However, in addition to classical fibroblasts and myofibroblasts be able to be derived from adipocytes with high plasticity. This process of interconversion of adipocyte and myofibroblast fates has been described as adipocyte mesenchymal transition (AMT), marked by the downregulation of adipogenic markers and the acquisition of a mesenchymal phenotype (69). AMT is involved in various pathophysiological processes such as wound healing, scleroderma, and cancer (70, 71). Moreover, the ability of adipocytes to transdifferentiate into a fibroblast-like phenotype was greatly enhanced under the challenge of HFD. The presence of AMT in the pathological microenvironment of adipose tissue in obesity exacerbates fiber remodeling in the extracellular matrix.

#### 2.6.1 ADSCs to myofibroblasts

ADSCs can be transformed into myofibroblasts under the regulation of specific cytokines. ADSCs mainly exist in the stromal vascular part of adipose tissue, and their surface expressions are CD34^+^, CD45^-^, CD31^-^ and Scal^+^, which have the potential for multidirectional differentiation similar to bone marrow mesenchymal stem cells (72). Its biological behavior is largely determined by the surrounding microenvironment (73). Under the challenge of the high-fat diet, the expression of cell proliferation marker cyclin D1 in ADSCs was up-regulated, which greatly enhanced their proliferation ability and promoted many aggregations in its fibrous region (74). At the same time, it has been shown that ADSCs isolated from obese mice are significantly enhanced in their ability to secrete extracellular matrix components and acquire a myofibroblast-like phenotype (75). Even, fibroblasts derived from ADSCs produced more extracellular matrix and migrated faster than primary skin fibroblasts (76). This may be due to the abundant TGF-β in the adipose tissue of obese mice. TGF-β is generally considered to be a master regulator of tissue fibrosis. By binding to type I and type II receptors, it promotes phosphorylation of Smad2/3 and enhances ECM deposition (77). Increased TGF-β in adipose tissue promotes the expression of recombinant integrin alpha 5 (ITGA5) and myocardin-related transcription factor (MRTFA). Among them, MRTFA is an important pathogenic factor of AMT transformation (78).

The platelet-derived growth factor receptor alpha (PDGFRα) gene in ADSCs is a major regulator of AMT transformation in obesity. Marcelin et al. defined two sub-populations under the cell population based on the level of the cluster of differentiation 9 (CD9) expression. Among them, the low expression of PDGFRα^+^CD9 cells was rich in adipogenesis and lipid metabolism-related genes, like the well-known ADSCs. In PDGFRα^+^CD9 high cells, the expression of transcription factors promoting adipogenesis was low, while the expression of TGF-β signaling mediating pro-fibrosis and genes involved in ECM synthesis were highly expressed. In the mediation of PDGF signaling, the two cell subpopulations were out of balance. PDGFRα^+^CD9 low cells transformed into PDGFRα^+^CD9 high fibrophilic phenotype, and the expression of fibrosis markers increased hundreds of times. In addition, there was an increased frequency of CD9-high relative to low-expressing CD9 progenitors and more severe omental adipose tissue fibrosis in severely obese subjects (79–81). This evidence suggests that PDGFRα is an important regulator of cell differentiation direction in the early fibroblast-adipocyte lineage, promoting myofibroblast proliferation and differentiation at the expense of adipocyte production.

Other genes related to AMT in ADSCs have also been reported. LY6C^+^ PDGFRβ^+^ ADSCs, also known as fibro-inflammatory progenitors (FIPs), are potential contributors to fibrosis. mRNAs for proinflammatory cytokines and extracellular matrix components were more abundant in FIPs compared to normal ADSCs. In addition, FIPs exhibit some anti-lipogenic ability. It is not only insensitive to adipogenic stimulation itself but also can release secreted factors to inhibit the differentiation of adipocytes from ASDCs (82). Further studies showed that fine-tuning mitochondrial function was a key regulator of progenitor fate and function in white adipose tissue (83). This reminds us that ADSCs are heterogeneous cell populations with different adipogenic potentials, and it is expected that more characteristic molecular markers can be identified in the future to predict potential differentiation trajectories.

#### 2.6.2 Adipocytes to myofibroblasts

At first, mature adipocytes were thought to be terminally differentiated cells that could not proliferate. In 1986, When Sugihara et al. cultured adipocytes using the ceiling culture method, they found that adipocytes took on a fibroblast-like appearance and named this cell morphology dedifferentiated adipocytes (84). With further studies of adipocyte plasticity, it was found that dedifferentiated adipocytes (DFAT) not only resembled fibroblasts morphologically but also had altered gene expression during dedifferentiation (85). Genes related to adipogenesis and mitochondrial activity were downregulated while lipid droplets were rapidly secreted. Dedifferentiated adipocytes have up-regulated expression of genes associated with cell renewal and reprogramming, exhibiting stem cell-like properties such as certain proliferative capacity and the ability to re-differentiate into different cell lineages. *In vitro* differentiation experiments show that adipocytes were driven by transforming growth factor β to preferentially undergo fibrogenic differentiation (86). Through pulse-chase lineage tracing of mouse mature adipocytes. Zhang et al. confirmed *in vivo* that dedifferentiated mature adipocytes possess certain proliferation and redifferentiation potential and can transdifferentiate in response to bleomycin stimulation for myofibroblasts. The transdifferentiation of dermal adipocytes into myofibroblasts is essential for the repair of skin wounds. Furthermore, using single-cell RNA sequencing (scRNA-seq), they found that adipocytes first dedifferentiate into PDGFRα^+^ preadipocytes. Thus, the transdifferentiation of mature adipocytes into myofibroblasts may occur through a two-step process of dedifferentiation back to ADSCs and then differentiation into myofibroblasts (69, 70). However, there is evidence that the transformation between fibroblasts and adipocytes is reciprocal (87–89). In other words, AMT is a double-edged sword in the process of tissue fibrosis. It is not only an additional pathogenic source of myofibroblasts but also a key part of reversing fibrosis.

## 3 The influence of adipose tissue fibrosis on other diseases

**Figure 3:**
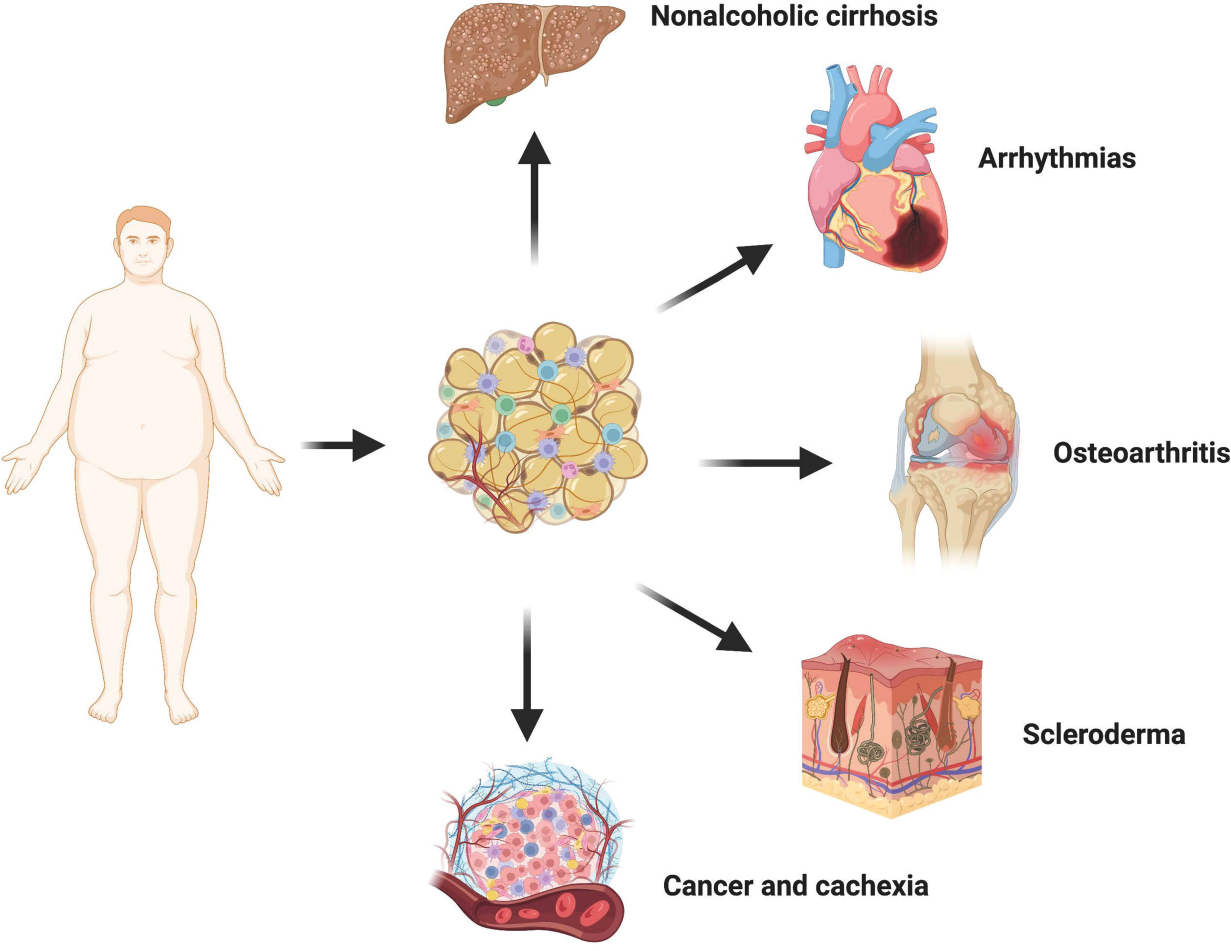
Crosstalk between adipose tissue and other organs. In the state of obesity, adipocytes undergo transdifferentiation into myofibroblasts via the release of various fibrophilic cytokines, thereby promoting local fibrosis of the adipose tissue. The presence of fibrotic adipose tissue not only significantly restricts the hypertrophy and proliferative adaptability of adipocytes themselves but also exerts a detrimental influence on neighboring tissues and organs through direct infiltration. Consequently, there is an increased risk and severity associated with non-alcoholic liver cirrhosis, cardiovascular disease, osteoarthritis, cancer, and scleroderma.

Adipose tissue is widely distributed throughout the human body and exhibits close interactions with various tissues and organs, rendering it a crucial biosensor for metabolic health (**Figure 3**). Different adipose tissue reservoirs possess distinct morphologies and functions, enabling them to perceive and respond to external signals from the systemic circulation and local microenvironment of other organs. In cases of obesity, adipocytes release fibrophilic adipokines, proinflammatory factors, and undergo transdifferentiation into myofibroblasts within the fat pad itself, significantly impeding the adaptability necessary for adipocyte growth and proliferation. Consequently, damaged fibrofatty tissue can directly infiltrate neighboring tissues such as the heart, bones, and joints leading to detrimental effects. Moreover, it can also transmit information to metabolically active organs through paracrine effects, interference in intercellular communication pathways, as well as abnormal accumulation of lipid components thereby elevating the risk of fibrosis development. Extensive literature has documented lipotoxicity observed in diverse human diseases along with experimental animal models. Lipotoxicity is the abnormal accumulation of toxic lipids in cells primarily caused by FFA. The mechanisms of lipotoxicity involve various cellular processes, including organelle damage and activation of intracellular signaling pathways. Damaged cells are prone to apoptosis or necrotic cell death, which releases many inflammatory cytokines and fibrotic mediators to further aggravate tissue fibrosis (90). In addition, in scleroderma and cancer, the loss of large amounts of intradermal fat is often associated with an adverse outcome. Therefore, fibrotic adipose tissue is not just localized damage to adipose tissue. It can also participate in the occurrence and development of a variety of diseases through strong cell-autonomous and paracrine effects, and ultimately produce systemic effects.

### 3.1 Osteoarthritis

Osteoarthritis (OA) is a complex disease with multiple contributing factors, characterized by pain, joint dysfunction, and chronic disability. The pathogenesis of this condition involves the interplay between increased biomechanical joint load due to obesity or systemic inflammation and metabolic dysfunction (91, 92). Notably, obesity induces fibrosis in the subchondral fat pad, significantly elevating the susceptibility to osteoarthritis (93). Moreover, fibrosis of the fatty pad beneath the patella seems to be a common feature of most osteoarthritis. During total knee replacement, Harasymowicz et al. biopsied the synovial and subpatellar fat pad of the knee in patients with end-stage osteoarthritis: adiponectin expression in synovial adipose tissue was significantly reduced in obese patients, showing marked fibrosis and macrophage infiltration, which may affect the nutrient supply to the articular cartilage (94). In addition, the fat pad under the patella, as an endocrine organ, in combination with other pathogenic factors such as mechanical loading, releases inflammatory mediators and adipokines such as IL-1, interleukin-13 (IL-13) and leptin into the knee joint, exacerbating the pathological damage of osteoarthritis (95–97). In contrast, transgenic mice with lipodystrophy exhibited diminished spontaneous knee injury and pain-related behaviors, along with resistance to high-fat diet-induced proinflammatory tendencies. Conversely, their susceptibility to osteoarthritis could be reinstated through adipose tissue transplantation (92).

### 3.2 Nonalcoholic cirrhosis

Non-alcoholic cirrhosis (NAFLD) is the most common chronic liver disease worldwide, mainly affecting people with obesity and type 2 diabetes. The reduction of central obesity in patients, particularly the longitudinal decrease in subcutaneous adipose tissue (SAT) and VAT volume, contributes to the histological amelioration of NAFLD (98). The study of Leven et al. showed that collagen deposition in visceral white adipose tissue (vWAT) of NAFLD patients was significantly increased (99). This indicates that there is a certain relationship between the fibrosis of adipose tissue and the occurrence and development of NAFLD, which may be related to the increase of lipid components. During obesity, the release of adipose tissue into circulation surpasses the liver’s inherent capacity to buffer lipids. Furthermore, obesity diminishes the production of Neuregulin 4, an adipokine that enhances hepatic lipid metabolism (100). Many types of lipid components have been shown to cause liver damage, including FFA, triglyceride (TG), free cholesterol (FC), etc. Injured hepatocytes released numerous inflammatory cytokines and fibrotic mediators, further aggravating liver pathology (101). Recently, Yu et al. showed that lipid accumulation-induced hepatocyte senescence activates hepatic stellate cells through the nuclear factor erythroid 2-related factor 2 (Nrf2)-antioxidant response element pathway. Under FFA-treated conditions, hepatocytes significantly increased the activation of co-cultured primary hepatic stellate cells (HSCs) and the expression of pro-fibrotic molecules while senescent (102). Dysregulation of adipokines further exacerbates liver injury in obesity. Cysteine-like protein 1 secreted by white adipose tissue (WAT) aggravates liver injury and inflammation in a mouse model of nonalcoholic steatohepatitis (NASH) under high-fat diet conditions by activating Toll-like receptor 4 (103). Moreover, hypolipinemia associated with obesity also contributes to the development of hepatic steatosis, fibrosis, and hepatocellular carcinoma (104).

### 3.3 Cancer and cachexia

Epidemiological evidence strongly indicates a significant correlation between excess weight or obesity and the development of numerous types of cancer (105). Gene expression associated with cancer progression is profoundly affected by obesity, and adipocytes from obese individuals create more favorable conditions for tumor formation. Data suggest that breast cancer incidence and metastatic risk are significantly higher in populations with higher BMI (106). It may be that factors secreted by adipose tissue, particularly in obese individuals, alter the transcriptome profile of breast cancer cells and promote reprogramming of cancer cell metabolism to produce a more aggressive phenotype (107, 108). Obesity induces upregulation of aromatase expression and downregulation of sex hormone binding globulin levels in adipose tissue, resulting in elevated free estrogen content. Consequently, this stimulates endometrial hyperplasia and augments the risk of cancer (109, 110). Leptin facilitates the proliferation and functional activation of endometrial cancer cells by modulating JAK2/STAT3, MAPK/ERK, and PI3K/AKT signaling pathways (111). Furthermore, obesity-generated fibrotic fat microenvironment also contributes to increased overall tumor or cancer fibrosis levels. Incio et al. found that pancreatic ductal adenocarcinoma (PDAC) in obese patients presented with hypertrophic adipocytes and more pronounced ECM deposition (112). Dense connective tissue hyperplasia compromises blood perfusion and poses a huge obstacle to the delivery and efficacy of chemotherapeutic drugs, leading to poorer treatment outcomes.

Obesity is not only a risk factor for cancer development but is also directly associated with poor prognosis in multiple tumor types. About 80% of cancer patients in advanced stages develop cachexia, characterized by continued uncontrolled weight loss, which directly leads to death in 22-40% of patients with end-stage cancer (113). Reduction in fat cell size, rupture of the capsule, and excessive deposition of extracellular matrix were observed in both patients and mouse models of cachexia. Even more interesting, Myofibroblasts in fibrotic regions often surround fat cells (114). Compared with normal fibroblasts, CAF has heterogeneity and high plasticity (115). ADSCs are an important cell source of CAF, and this process is closely related to the activation of the Wnt signaling pathway (116). In addition, it was noted that overweight or obese patients had higher levels of ADSCs circulating in their blood compared to cancer patients with lower body weight (117). Taken together, it is reasonable to assume that increased stromal stiffness in adipose tissue is an important mediator of cancer onset and progression. And inhibiting the excessive deposition of stroma by blocking the transformation of ADSCs to CAF may be a very promising treatment for fibrosis-related cancers.

### 3.4 Arrhythmias and heart failure

The smooth transmission of cardiomyocyte potential depends on the special electrophysiological and structural characteristics of heart tissue, abnormal cardiac excitation will lead to arrhythmia. Atrial fibrillation is the most common type in clinical practice and has a high risk of death due to the risk of hemodynamic abnormalities and thromboembolism (118). Over the years, a variety of risk factors for arrhythmias have been identified, among which an increase in the number of non-excitable cells due to abnormalities in cardiac tissue remodeling has been extensively studied. Obesity generally leads to more frequent and persistent atrial fibrillation, which may indicate that fiber remodeling of extra-cardiac adipose tissue may play a role in promoting myocardial fibrosis (119, 120).

Extracardial adipose tissue (EAT) is in direct contact with the atria and shares a common blood supply with the myocardium. To mitigate lipotoxicity, epicardial adipose tissue (EAT) demonstrates enhanced rates of FFA uptake compared to subcutaneous adipose tissue (SAT), and exhibits heightened sensitivity towards variations in dietary lipid content. While the heart predominantly relies on lipids as metabolic substrates, excessive lipid accumulation can give rise to detrimental consequences. Excessive deposition of lipids may lead to severe arrhythmogenic complications (121). Simultaneously, recent experiments have shown that there is a close relationship between fibrofatty infiltration and arrhythmia caused by myocardial fibrosis (122, 123). The infiltration of fat cells itself interferes with the normal conduction of cardiomyocyte potential (124). Extracardiac adipose tissue has been proven to secrete fibrophilic mediators, such as connective tissue growth factor CTGF, TGF-β, and Activin A to promote myocardial fibrosis (125, 126). Moreover, epicardial adipose tissue serves as a localized indicator of systemic inflammation in individuals with obesity and has the capability to secrete diverse inflammatory mediators, including IL-6 and TNFα (127). Under the combined drive of these factors, the pericardial mesenchymal stem cells can migrate to the ventricular muscle and transform into fibroblasts, promoting the fibrotic remodeling of the myocardium (128). Infiltrated adipocytes, fibroblasts, and myofibroblasts can form gap junctions with adjacent cardiomyocytes through connexin, affecting normal electrophysiological conduction of cardiomyocytes.

At the same time, it has been thought that the effect of obesity on heart failure is achieved by increasing the load of the heart through hemodynamics. However, most obese patients have only a slight increase in heart volume, most of which is ejection fraction reserved heart failure (HFpEF), which is characterized by fibrotic failure of the cardiac microvascular supply and limited expansion of the heart (129, 130). Most significantly, bariatric surgery effectively mitigates ventricular repolarization heterogeneity in obese patients (131).

### 3.5 Scleroderma

Scleroderma (SSc) is marked by excessive deposition of ECM proteins caused by abnormal activation of myofibroblasts, which mainly affects the skin and blood vessel walls, not only causing inconvenience to patients’ daily life but also potentially causing fatal organ dysfunction (132). Dysfunctions of adipocytes and abnormal secretion of adipokines are key events in the progression of scleroderma fibrosis. First, skin fibrosis in SSc is often accompanied by significant loss of intradermal fat. Moreover, myofibroblasts in scleroderma have been shown to originate at least in part from subdermal fat cells (133). More importantly, in chronological terms, atrophy of intradermal adipose tissue usually precedes myofibroblast accumulation and subsequent skin thickening (86). Further studies showed that ADSCs produced AMT in bleomycin-induced fibrotic skin, exhibiting the characteristics of myofibroblasts (134). This may be caused by the significantly up-regulated nuclear receptor corepressor (NcoR) signal in SSc. NCoR is a negative regulator of gene expression. It recruits histone deacetylation (transcriptional inhibition) enzymes to the DNA promoter region, and its main function in adipose tissue is to inhibit PPAR-γ transcriptional activity (135). Injection of normal ADSCs or fat transplantation into scleroderma patients has achieved good results in softening skin and alleviating fibrosis in animal models and clinics (136, 137). This evidence conveys a valuable message that the loss of adipose tissue in scleroderma is not merely a pathological phenomenon, but a direct contribution to skin fibrosis.

## 4 Anti-fibrotic strategies targeting adipose tissue fibrosis

Imbalance in energy homeostasis causes adipose tissue fibrosis, which exacerbates the progression of obesity-related fibrotic disease. Therefore, focusing on maintaining the healthy expansion of adipocytes and preventing excessive deposition of extracellular matrix components is an attractive proposition against pathological fibrotic remodeling. Current major strategies focus on altering the function of white adipose tissue, targeted therapy, and dietary therapy.

### 4.1 Dietary therapy

Factors that predispose to obesity in modern lifestyles include longer periods of food intake and shorter periods of fasting. However, it is unrealistic to expect overweight patients to strictly adhere to daily calorie restrictions (DR). Comparatively, intermittent eating (IF) is widely adopted as a more operational dietary regimen involving repeated and regular energy restriction, resulting in multiple health benefits. Meanwhile, it was found that IF mediates adaptive tissue remodeling of WAT to prevent HFD-induced adipose tissue inflammation and fibrosis (138). In addition to reducing energy intake, supplementing some nutrients with special functions is also an effective means to resist WAT fibrosis. Berberine, present in a variety of Chinese herbal plants, has been shown to have therapeutic potential in the treatment of diabetes and dyslipidemia. As a natural anti-inflammatory compound, berberine reverses signaling and controls HFD-induced macrophage infiltration and polarization. Beyond that, berberine alleviates adipose tissue fibrosis by inducing AMP-activated kinase signaling in high-fat diet-induced obese mice (139). Recently, in a randomized controlled trial, Lontchi-Yimagou et al. found that increasing vitamin D supplementation within the normal range had a beneficial effect on suppressing adipose tissue inflammation and fibrosis in obese subjects. This phenomenon was also confirmed in adipocyte-specific vitamin D receptor knockout (Ad-VDR KO) mice (140). Similarly, oral administration of the antioxidant vitamin E has also been reported to increase oxidative stress and reduce collagen deposition in vWAT in obese mice, thereby increasing the lipid storage capacity of adipocytes and reducing obesity-related lipotoxicity (141). Unfortunately, it is unclear how long the effects of oral nutritional supplementation treatments can be sustained.

### 4.2 Medical treatment

#### 4.2.1 Promotes WAT browning

Unlike WAT, which is primarily used for energy storage, BAT converts energy into heat by decoupling-protein uncoupling protein 1 (UCP-1) (142). Meanwhile, WAT deposits contain polyocular cells that express UCP-1, called beige cells, which are stimulated by the browning process when exposed to cold or other stimuli. In recent years, more and more studies have shown that the browning and fibrotic remodeling of adipose tissue are two opposing processes. MRTFA-deficient mice are resistant to HFD-induced adipocyte overexpansion and have increased numbers of beige adipocytes in their WAT (143). Further studies revealed that positive regulatory domain 16 (PRDM16) that controls BAT development could promote β-hydroxybutyrate (BHB) secretion by driving fatty acid oxidative metabolism, which rescued adipose expressing HIF1α or TGFβ-treated PDGFRα^+^ ADSCs cell differentiation potential (144). Interestingly, the anti-fibrotic capacity of BAT may also act in a UCP-1-independent manner. Activation of the PRDM16 transcriptional complex effectively suppressed adipose tissue fibrosis through direct interaction with GTF2IRD1 (145). And it is worth noting that many browning-inducing drugs have been shown to have potent anti-fibrotic effects at the same time (**Table 1**). Therefore, induction of browning of white fat is a potential therapeutic strategy against HFD-induced adipose tissue fibrosis and related dysfunction.

**Table 1:** Antifibrotic drugs or factors associated with browning.

Drug or cytokines	Pathway	Function	Reference
PPAR-γ agonists	PPAR-γ↑→FGF-21↑ PPAR-γ↑→TGF-β1↓	1. Differentiation of bone marrow mesenchymal stem cells into fibroblasts↓ 2. Lipid metabolism and browning of WAT↑	(146–148)
NOTUM	NOTUM→Wnt3a↓→TGF-β↓	1. Expression of fibrosis-related genes↓ 2. Expression of thermogenesis-related genes in WAT and BAT↑ 3. Differentiation of BAT↑	(149)
Metformin	AMPK↑, PPAR-γ↑ ROS/NF-KB↓→IL-6, TNF-α, TGF-β↓ HIF1α↓, HMGB1↓	1. Expression of various collagen genes↓ 2. Excessive ECM deposition↓ 3. Browning of WAT↑	(150–153)
Thiazolidinedione	PPAR-γ↑→α-SMA↓	1. Expression of α-SMA in fibroblasts↓ 2. Browning of WAT↑	(154, 155)
SRT1720	Sirt1↑→TGF-β1/CTGF↓ Sirt1↑→HIF1α/GLUT1↓	1. EMT and collagen deposition↑ 2. Oxidative stress levels↓ 3. Fatty acid oxidation and glucose metabolism↑	(156, 157)
Roscovitine	TGF-β↓→P38 MAPK↓→α-SMA↓	1. EMT and TGF-β signaling pathways↓ 2. Browning of WAT↑	(158)
Resveratrol	AMPK↑→Sirt1↑→HIF-1↓	1. Activation of HIF-1α↓ 2. Browning of WAT↑	(159)

↑ indicates the up-regulation of the signaling pathway or histological changes, while ↓ indicates the down-regulation of the signaling pathway or histological changes.

#### 4.2.2 Enhance lipid metabolism

Dysfunction of adipocytes begins with excessive lipid accumulation, so accelerating the metabolic function of adipose tissue and improving its lipid buffering capacity can prevent the occurrence of obesity-related fibrotic comorbidities. As a key site of oxidative phosphorylation, mitochondria play a complex and important role in maintaining the metabolic homeostasis of adipocytes. Serine/thionine kinase 25 (Skt25) protects against diet-induced adipose tissue fibrosis by regulating mitochondrial activity in adipose tissue (160). At the same time, there is evidence that the impaired metabolic function of adipocytes in the obese state is closely related to the down-regulation of miR-30a. Overexpression of miR-30a in adipose tissue allows smooth expansion of adipose tissue in a manner that preserves insulin sensitivity (161). Furthermore, proteomic analysis revealed that miR-30a restores WAT homeostasis by targeting plasminogen activator inhibitor 1 (PAI-1) to limit the pro-fibrotic program.

### 4.3 Targeted therapy

CD248 (endothelin/tumor endothelial marker 1) is a type I transmembrane glycoprotein that is most abundantly expressed in human mature white adipocytes and significantly correlated with body mass index (BMI). Petrus et al. suggest that CD248 acts as an adipocyte sensor in the microenvironment and may mediate the abnormal biological behavior of adipocytes. Transcriptome analysis of human WAT revealed that CD248 expression was positively correlated with inflammation, hypoxia, and ECM remodeling. CD248 deficiency protects against high-fat diet-induced WAT dysfunction. Besides, adipocyte-specific knockout exhibited a greater therapeutic effect than systemic knockout. Notably, these improvements were also achieved in mice following the onset of obesity. Therefore, the rescue of WAT fibrosis by selectively reducing CD248 expression in adipocytes is an attractive target for future drug development (162). However, the specific molecular mechanism by which CD248 acts has not been fully described. Therefore, a better understanding of the signaling pathways downstream of CD248 is critical for adipocyte-specific targeted therapy.

## 5 Conclusion

In general, in the state of obesity, fat cells are dysfunctional and interact with immune cells to produce a fibrogenic cellular microenvironment, which affects the plasticity of white adipose tissue and the normal function of surrounding organs and promotes the occurrence and development of a variety of fibrosis-related diseases. Specifically, adipocytes promote fibrosis by secreting inflammatory mediators, and adipokines and changing their fate to become myofibroblasts. This suggests that when studying many systemic diseases associated with fibrosis, it is time to focus on the nearest reservoir of adipose tissue. At present, the use of adipose tissue to regulate fibrosis has great untapped potential, but due to the complexity of the dialogue between adipose cells and fibroblasts, there is still a lot of work to be done to figure out the specific mechanism of adipose cells involved in regulating the process of tissue fibrosis.

First, most studies of adipocyte secretory factors promoting fibrosis have been limited to animals or *in vitro*. The specific signaling pathway that promotes fibrosis is still unknown. Secondly, further studies of the AMT are needed to provide more solid evidence to confirm whether myofibroblasts produced by fat cells are a one-step or multi-step process. Besides, it is expected to decipher the key aggregation points downstream of various signaling pathways in the adipocyte-myofibroblast transition process, to realize the controllability of transformation. Finally, inducing differentiation of myofibroblasts into brown adipocytes is a feasible strategy for antagonizing fibrosis. However, it should be noted that most of the anti-fibrosis drugs mentioned in this paper have a wide range of targets, so the side effects of drugs should not be underestimated. Therefore, it is urgent to find effective brown inducers or inducers for specific adipose tissue. At the same time, another great challenge is how to effectively complete the transformation of myofibroblasts into brown fat cells under pathological conditions. Because in an obese state, cells are much less sensitive to browning agonists. Moreover, there is a lack of drugs that can effectively increase the expression of brown adipose tissue in humans. We believe that if these mechanisms can be elucidated through subsequent experimental studies and brown fat cells are indeed effective in antagonizing fibrosis, regulating the biological behavior of fat cells will be a new option for treating many fibrosis-related diseases.

## Author contributions

QZ: Writing – original draft. CL: Writing – review & editing, Data curation, Methodology. FL: Methodology, Writing – review & editing. YL: Writing – review & editing. JC: Methodology, Writing – review & editing. JG: Funding acquisition, Methodology, Writing – original draft, Writing – review & editing.

## Glossary

**Table:**

ECM	Extracellular matrix
ADicerKO	Adipose tissue-specific knockout of the miRNA-processing enzyme Dicer
Ad-VDR KO	Adipocyte-specific vitamin D receptor knockout
AdipoR1	Adiponectin Receptor 1
ADSCs	Adipose-derived stem cells
AKT	Protein kinase B
AMPK	Amp-activated protein kinase
AMT	Adipocyte mesenchymal transition
ATF2	Activating transcription factor 2
BAT	Brown adipose tissue
BHB	β-hydroxybutyric acid
BMI	Body mass index
CAF	Cancer-related fibroblasts
CD248	Endothelin/tumor endothelial marker 1
CD9	Cluster of differentiation 9
CLS	Crown-like structure
COL6-α3	Recombinant Collagen Type VI-Alpha 3
CTGF	Connective tissue growth factor
DFAT	Dedifferentiated adipocytes
DIO	Diet-induced obesity
DPT	Dermatopontin
DR	Daily calorie restriction
EAT	Extracardial adipose tissue
EHMT1	Euchromatic histone-lysine N-methyltransferase 1
EMT	Epithelial-mesenchymal transition
ERK	Extracellular regulated protein kinases
Evs	Extracellular vesicles
FC	Free cholesterol
FFA	Free fatty acid
FGF-21	Fibroblast growth factor-21
GLUT1	Glucose transporter 1
GPCR	G-protein-coupled receptor
HFD	High-fat diet
HFpEF	Ejection fraction reserved heart failure
HIF-1	Hypoxia-inducible factor-1
HMGB1	High mobility group box 1
HSCs	Hepatic stellate cells
IFN-γ	Interferon-γ
IL-1	Interleukin-1
IL-13	Interleukin-13
IL-6	Interleukin-6
ILC1s	Group 1 innate lymphoid cells
ITGA5	Recombinant integrin alpha 5
LOX	Lipoxygenase
MCP-1	Macrophage chemoattractant protein-1
METS	Metabolic syndrome
Mincle	Macrophage-inducible C-type lectin
MRTFA	Myocardin-related transcription factor A
NAFLD	Non-alcoholic cirrhosis
NASH	Nonalcoholic steatohepatitis
NcoR	Nuclear receptor corepressor
Nrf2	Nuclear factor erythroid 2-related factor 2
OA	Osteoarthritis
PDGF	Platelet-derived growth factor
PDGFR-α	Platelet-derived growth factor receptor-alpha
PI3K	Phosphatidylinositol 3 kinase
PPAR-α	Peroxisome proliferator-activated receptor-α
PPAR-γ	Peroxisome proliferator-activated receptor-γ
PRDM16	Positive regulatory domain containing 16
p-STAT3	Phosphorylated signal tranducers and activators of transcription
p38 MAPK	P38- mitogen-activated protein kinases
ROS	Reactive oxygen species
SAT	Subcutaneous adipose tissue
scRNA-seq	Single cell RNA sequencing
Smad3	Small Mothers Against Decapentaplegic
SSc	Scleroderma
Skt25	Serine/thionine kinase 25
SVFs	Stromal vascular fractions
T2D	Type 2 diabetes
TG	Triglyceride
TGF-β	Transforming growth factor-β
TNFα	Tumor necrosis factor-α
TRAMP	Tyrosine-rich acidic mechanism protein
UCP-1	Uncoupling protein-1
vWAT	Visceral white adipose tissue
WAT	White adipose tissue
α-SMA	α-smooth muscle actin
